# 非小细胞肺癌组织中ZO-1的表达及其临床意义

**DOI:** 10.3779/j.issn.1009-3419.2011.02.08

**Published:** 2011-02-20

**Authors:** 晔恺 王, 吉航 周, 芳 曾, 燕燕 黄, 世权 周, 晓光 刘

**Affiliations:** 316004 舟山，浙江省舟山医院细胞分子生物学实验室 Laboratory of Molecular Biology, Zhoushan Hospital, 316004 Zhoushan, China

**Keywords:** ZO-1蛋白, 肺肿瘤, 免疫组织化学, ZO-1 protein, Lung neoplasms, Immunohistochemistry

## Abstract

**背景与目的:**

已有的研究表明紧密连接蛋白-1（zonula occluden-1, ZO-1）表达和肿瘤细胞的生长和转移存在密切联系。本研究旨在探讨非小细胞肺癌（non-small cell lung cancer, NSCLC）组织中ZO-1表达的临床意义。

**方法:**

应用实时荧光定量PCR、Western blot和免疫组化检测101例NSCLC癌组织及癌旁组织中ZO-1 mRNA和蛋白表达，以61例肺部良性病变患者作为对照。

**结果:**

ZO-1 mRNA在癌组织和肺良性病变组织间差异具有统计学意义（*P*＜0.01）。Western blot显示：ZO-1蛋白在癌组织、癌旁组织、肺良性病变组织的相对表达量之间均存在明显差异（*P*＜0.01或*P*＜0.05）。免疫组化显示：ZO-1平均光密度在癌组织、癌旁组织和肺良性病变组织中分别为69.55± 17.13、246.39±83.15、330.93±72.44，组间差异均具有统计学意义（*P*＜0.01）。伴有淋巴结转移与不伴淋巴结转移肺癌组织ZO-1 mRNA表达水平比较有显著性差异（*t*=-5.07, *P*＜0.01），肺癌患者术后2年内生存组和2年内死亡组间ZO-1 mRNA表达水平比较差异具有统计学意义（*t*=5.61, *P*＜0.01）。

**结论:**

ZO-1 mRNA和蛋白在NSCLC癌组织中表达降低，并与淋巴结转移的术后生存有密切关系。

## Abstract (en)

**Background and objective:**

It has been proven that the expression of ZO-1 (zonula occluden-1) was correlated with the growth and metastasis of cancer. The aim of this study is to investigate the relationship between *ZO*-*1* gene expression and clinicopathologic parameters and the survival rates in the patients with non-small cell lung cancer (NSCLC).

**Methods:**

The expression levels of *ZO*-*1* gene in 101 patients with NSCLC and lung tissues of 61 patients with benign lung disease were detected by real-time PCR, Western blot and immunohistochemical techniques.

**Results:**

There was a significant statistic difference of ZO-1 mRNA expression levels between carcinoma group and control group (*P* < 0.01). In carcinoma group, adjacent group and control group, ZO-1 protein expression levels were with significantly statistic difference between each other (*P* < 0.01 or *P* < 0.05). There was a significantly statistic difference between ZO-1 mRNA expression levels in survival during 2 years follow-up group and death during 2 years follow-up group (*t*=-5.61, *P* < 0.01).

**Conclusion:**

ZO-1 is a valuable predictive marker for NSCLC patients.

紧密连接蛋白-1（zonula occluden-1, ZO-1）是一种与紧密连接有关的蛋白，参与维持和调节上皮屏障功能，常作为观察各种组织紧密连接屏障功能和通透性功能的指标，如血脑屏障^[1]^、肠上皮细胞^[2]^等。为探讨ZO-1与非小细胞肺癌（non-small cell lung cancer, NSCLC）临床及病理特性的关系，本研究通过实时荧光定量PCR、Western blot、免疫组化检测101例NSCLC患者癌组织和癌旁组织的ZO-1 mRNA和蛋白表达，探讨其与肿瘤分期、淋巴结转移、预后等临床指标的相关性。

## 1 材料与方法

### 1.1 对象

收集舟山医院2006年1月-2008年2月101例NSCLC患者（包括腺癌41例，鳞癌43例，细支气管肺泡癌17例）癌组织和癌旁组织（距离癌组织2 cm）标本，男性65例，女性36例，年龄（42-80）岁，平均年龄（61.20± 8.51）岁。以61例肺部良性病变为对照（炎性假瘤33例，机化性肺炎18例，支气管扩张8例，结核球1例，错构瘤1例），男性40例，女性21例，年龄（41-82）岁，平均年龄（67.37±11.02）岁。诊断均经病理切片确认，置液氮罐中保存，取材为病理切片完成后所留组织，经舟山医院伦理学委员会批准并征得患者同意。

### 1.2 仪器和试剂

兔抗人ZO-1一抗购自Zymed Labs（WB和IHC通用型），兔抗人GAPDH一抗购自Abcam，SP-9000免疫组化试剂盒购自中杉金桥，细胞裂解液、BCA蛋白浓度测定试剂盒、PVDF膜、HRP标记山羊抗兔二抗均购自碧云天，Trizol购自Invitrogen，pMD18载体试剂盒和荧光定量PCR试剂盒（Syber Green Ⅰ）购自Takara，荧光定量PCR引物（S: Sense; A: Antisense）：ZO-1-S：5’-GAGATGAACGGGCTACGC-3’，ZO-1-A：5’-GAGACTGCCATTGCTTGG-3’，扩增产物230 bp；GAPDH-S：5’-GACCTGACCTGCCGTCTA-3’，GAPDH-A：5’-AGGAGTGGGTGTCGCTGT-3’，扩增产物148 bp；均由上海生工合成，荧光PCR仪为Roche Lightcycle，图像采集系统为Olypus-BX60，凝胶成像系统为美国Bio-Rad公司Universal Hood Ⅱ型，凝胶成像分析软件为Quantity One，匀浆器为德国IKA T10 basic型。

### 1.3 ZO-1实时荧光定量PCR

#### 1.3.1 ZO-1和GAPDH标准品构建

Trizol提取总RNA，鉴定完整性和纯度，取5 μg总RNA冰上逆转录成cDNA进行逆转录PCR，反应体系25 μL：其中DNA模板3 μL，10 μmol/ L ZO-1或GAPDH荧光定量PCR引物各1 μL，5×PCR缓冲液5 μL，加去RNA酶水补至25 μL，反应条件：95 ℃、5 min；94 ℃、30 s，53.7 ℃、30 s，72 ℃、1 min，30个循环；72 ℃、10 min。PCR反应产物经纯化后，加入连接缓冲液5 μL、纯化的目的片段4.5 μL、pMD18-T载体0.5 μL，低速离心后于16 ℃连接过夜，将连接液转化大肠杆菌DH5α后涂氨苄青霉素平板，挑取单菌落培养过夜，抽提其质粒通过PCR鉴定阳性重组子，送上海生工公司测序确证。

#### 1.3.2 实时荧光定量PCR

将重组质粒10倍4梯度稀释做为标准品，对cDNA进行Syber Green Ⅰ荧光染料嵌合法检测，反应体系参照试剂说明书，反应条件：95 ℃、5 min；94 ℃、30 s，53.7 ℃、30 s，72 ℃、60 s，40个循环；72 ℃、10 min，利用各自的标准曲线分别对样品中的目的基因和内参基因分别进行定量，肺组织中ZO-1 mRNA的相对表达量为*ZO*-*1*基因拷贝数/*GAPDH*基因拷贝数。

### 1.4 Western blot检测ZO-1蛋白表达

#### 1.4.1 肺组织蛋白提取

预冷PBS洗涤组织2次，将肺组织剪碎，匀浆，加入适量含PMSF的RAPI裂解液，冰上裂解30 min，4 ℃下12, 000 rpm离心15 min，取上清用BCA法测定蛋白浓度，分装后-80 ℃保存。

#### 1.4.2 Western blot

分别取总蛋白上清液24 μL，加6 μL 5×上样缓冲液混匀。用普通PCR仪煮沸10 min变性后取30 μg上样，进行SDS-PAGE电泳分离蛋白（12%分离胶，5%浓缩胶），转移至PVDF膜上（300 mA, 30 min），5%脱脂牛奶封闭非特异抗原2 h，按照蛋白质Marker切割PVDF膜，并分别加入1:2, 000稀释的兔抗人ZO-1多克隆抗体和1:500稀释的兔抗人GAPDH多克隆抗体，4 ℃过夜。PBS-T洗涤3次，每次10 min。再分别加入1:5, 000稀释的羊抗兔IgGHRP，室温震摇1 h，PBS-T洗涤3次，每次15 min。ECL系统显色，凝胶成像仪检测，采用Quaintity One分析软件进行条带分子量及灰度值的分析。

### 1.5 ZO-1免疫组化

石蜡切片脱蜡水化后，PBS洗3遍，3% H_2_O_2_浸泡切片10 min封闭内源性过氧化物酶，微波热处理（98 ℃, 10 min）修复抗原，切片置于湿盒中，小牛血清常温孵育1 h封闭非特异性抗原，吸取封闭液，加兔抗人ZO-1一抗（1:100稀释），4 ℃孵育过夜，孵育结束后吸去一抗，PBS-T洗片3次，加人HRP标记山羊抗兔二抗（1:200稀释），室温下孵育30 min，孵育结束后吸去二抗，PBS-T洗片3次，滴加DAB显色3 min-5 min，自来水冲洗切片终止反应，苏木素复染30 s，常规脱水、封片放干过夜，棕褐色为阳性着色。以PBS代替一抗作为阴性对照。显微镜下摄片，用Imagepro-Plus 6.0进行积分光密度值（integral optical density, IOD）测定并计算平均光密度（average of the optical densities, AOD），选定组织区域，通过AOD测定ZO-1蛋白的表达，其值越大，表达量越高。

### 1.6 生存随访

2年后采用记录在案的电话随访101例肺癌患者本人或家属，有6例失访，在访的95例患者中44例死于肺癌复发或远端转移引起的疾病，未有非正常死亡。

### 1.7 统计学分析

采用SPSS 13.0统计软件，用t检验单因素方差分析进行数据分析，以*P*＜0.05为差异具有统计学意义。

## 2 结果

### 2.1 肺癌患者癌组织、癌旁组织和肺良性病变肺组织中ZO-1 mRNA和蛋白

ZO-1 mRNA在癌组织、癌旁组织、肺良性病变肺组织中的相对表达量分别为0.99±0.47、1.10± 0.55、1.24±0.39，肺癌组织与肺良性病变肺组织之间比较具有显著性差异（*P*＜0.01）。ZO-1蛋白在癌组织、癌旁组织、对照组中的相对表达量分别为0.45±0.31、0.75± 0.26、0.87±0.39，癌组织和对照组之间、癌组织和癌旁组织之间的差异均具有统计学意义（*P*＜0.01），癌旁组织和对照组之间的差异亦具有统计学意义（*P*＜0.05）（图 1）。

**1:**
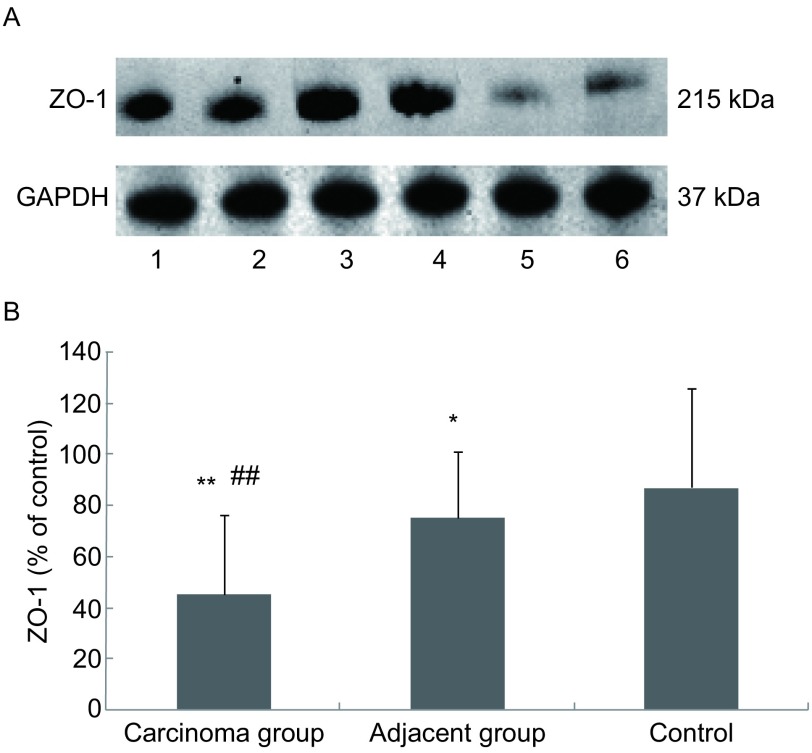
肺癌患者癌组织、癌旁组织和对照组中ZO-1蛋白表达。A：Wester blot检测ZO-1表达；B：柱状分析图表明ZO-1表达在癌组织、癌旁组织和对照组中的差异存在明显差异；泳道1-2：癌旁组织；泳道3-4：对照组；泳道5-6：癌组织；^*^：与对照组相比，*P*＜0.05；^**^：与对照组相比，*P*＜0.01；^##^：与癌旁组相比，*P*＜0.01。 Expression of ZO-1 protein in carcinoma tissue group, adjacent tissue group and control group. A: Expression of ZO-1 protein determined by Western blot; B: The densitometric analysis reveal that the levels of ZO-1 protein among the carcinoma tissue group, the adjacent tissue group and the control group were with significantly statistic difference; Lane 1-2: adjacent tissue group; Lane 3-4: control group; Lane 5-6: carcinoma tissue group; ^*^: compared with the control group, *P* < 0.05; ^**^: compared with the control group, *P* < 0.01; ^##^: compared with the adjacent tissue group, *P* < 0.01.

### 2.2 NSCLC患者癌组织和癌旁组织不同临床及病理因素中ZO-1 mRNA的表达

癌旁组织ZO-1 mRNA在淋巴结转移组和淋巴结未转移组中的差异具有统计学意义（*t*=-5.07, *P*＜0.01），2年生存组和2年死亡组中的差异具有统计学意义（*t*=5.61, *P*＜0.01）；癌组织中ZO-1 mRNA在2年生存组和2年死亡组的差异存在明显差异（*t*=3.81, *P*＜0.01）（表 1）。

**1:** NSCLC患者癌组织和癌旁组织不同临床及病理因素中的ZO-1 mRNA的表达 Expression of ZO-1 mRNA in different clinicopathologic parameters and the survial rates in carcinoma tissue and adjacent tissue of the patients with NSCLC

Clinicopathologic parameters	*n*	ZO-1 mRNA expression in carcinoma tissue	ZO-1 mRNA expression in adjacent tissue
Gender			
Male	65	0.95±0.41	1.05±0.51
Female	36	1.10±0.43	1.19±0.38
Pathologic type			
Gland carcinoma	41	0.99±0.35	1.13±0.56
Squamous carcinoma	43	0.93±0.27	1.08±0.37
Bronchioloalveolar carcinoma	17	1.13±0.47	1.07±0.48
Lymph node metastasis			
Positive	31	0.89±0.43	0.83±0.41^**^
Negative	70	1.03±0.29	1.22±0.33
Encroaches upon the pleural membrane			
Yes	49	0.93±0.48	1.05±0.35
No	52	1.04±0.45	1.14±0.49
Differentiated stage			
Poorly differentiated	64	1.01±0.44	1.12±0.41
Well differentiated	37	0.94±0.36	1.07±0.29
2-years survival			
Yes	51	1.21±0.26^**^	1.29±0.31^**^
No	44	0.87±0.33	1.02±0.38
^**^: compared with the other clinicopathologic parameters in the same tissue type, *P* < 0.01; Lymph node metastasis: Stage (N1 or N2 or N3) and M1[TNM]; Differentiated stage: well differentiated: pathological gradeI (bronchioloalveolar carcinoma included); poorly differentiated: pathological grade Ⅱ and Ⅲ.			

### 2.3 ZO-1蛋白在NSCLC患者肺组织中的免疫组化结果

在癌旁（图中为腺癌癌旁）和对照组织中，ZO-1沿着肺泡壁均匀分布；在癌组织中（图中为腺癌组织），ZO-1也沿着腺泡壁分布，但不完整，存在部分位置的缺失，腺癌细胞间的ZO-1呈低表达。ZO-1平均光密度在癌组织、癌旁组织和对照组中分别为69.55±17.13、246.39±83.15、330.93±72.44，组之间差异均具有统计学意义（*P*＜0.01）（图 2）。

**2:**
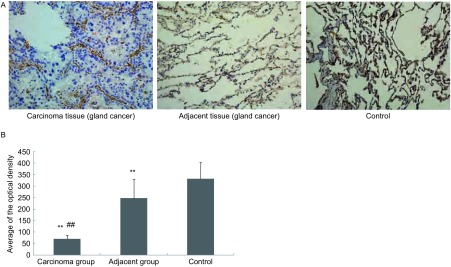
NSCLC患者肺组织中ZO-1免疫组化。A：ZO-1在肺组织中的免疫组化（SP，×20）；B：柱状分析图表明ZO-1表达在癌组织、癌旁组织和对照组中的差异存在显著统计学意义；^**^：与对照组相比，*P*＜0.01；^##^：与癌旁组相比，*P*＜0.01。 Immunohistochemical assessmen of ZO-1 in lung tissue of patients with NSCLC. A: Immunohistochemical assessmen of ZO-1 in lung tissue (SP, ×20). In adjacent tissue of gland cancer and control group, ZO-1 distributed through the alveolar walls completely. But in carcinoma tissue of gland cancer, ZO-1 distributed through the alveolar walls incompletely; there is a low expression of ZO-1 between the gland cancer cells; B: The densitometric analysis reveal that the averages of the optical densities of ZO-1 protein among the carcinoma tissue group, the adjacent tissue group and the control group were significant statistic different; ^**^: compared with the control group, *P* < 0.01; ^##^: compared with the adjacent tissue group, *P* < 0.01.

## 3 讨论

ZO-1属于膜鸟苷酸激酶MAGUK（membrance-associate guanylate kinase homologs, MAGUK）家族^[3]^，与ZO-2、ZO-3组成ZO-1/ZO-2/ZO-3复合体，该复合体有3个PDZ区，ZO-1通过其PDZ区与其它细胞连接蛋白如肌动蛋白^[4]^等相互连接。本研究通过将ZO-1和GAPDH构建到T载体作为标准品，利用Syber Green Ⅰ荧光染料嵌合法检测ZO-1和GAPDH mRNA的相对表达含量，通过检测显示癌组织中的ZO-1 mRNA和蛋白均低于对照组（*P*＜0.01）。由于ZO-1具有正常上皮细胞标志物的特性^[5]^，因此其mRNA的降低可能是肺癌早期发生的指标之一，这点在其它多种癌组织中已有报道：Fiorini等^[6]^发现化学药物DDT作用于ZO-1等影响到细胞耦合损伤，并推测可能在癌变中起作用；Orban等^[7]^通过RT-PCR发现原发性肝癌中出现ZO-1 mRNA相对于正常肝的表达降低。可见，ZO-1除了粘附和屏蔽功能外，还参与细胞耦合功能构建，其参与构建的紧密连接束（tight junction srtands, TJs）对上皮屏障功能有重要影响^[8]^。目前有报道证实，多种物质如糖原合酶激酶-3^[9]^、胰岛素样生长因子^[10]^等可通过作用于ZO-1影响TJs的构建或屏障功能。从免疫组化的结果来看，癌细胞间的ZO-1表达大量缺失，可能影响到癌组织中TJs的构建，使得肺部微环境有利于肿瘤细胞的原位增殖。

本研究还发现，在NSCLC癌组织中淋巴结远端转移和未转移组中的ZO-1mRNA未存在明显差异（*P*＞0.0 5），但两者在癌旁组织中却存在明显差异（*P*＜0.01），这可能是由于ZO-1主要表达于胞膜和细胞间质^[11]^，而肺组织淋巴系统内皮细胞间连接是由ZO-1等一系列紧密连接蛋白构建，ZO-1的低表达降低了周围微循环组织内皮细胞的紧密连接性，影响其栅栏屏障功能和免疫细胞的分布^[12]^，从而更有利于癌细胞通过通透性增高的组织扩散进入淋巴系统^[13]^。Tuomi等^[14]^发现蛋白激酶Cepsilon调控alpha 5-ZO-1复合体控制肺癌细胞迁移片层的形成。Zen等^[15]^发现在食管鳞癌患者组织中*PARD3*基因的低表达可干扰ZO-1的胞间位置，并和淋巴结转移存在相关性。Ohtani等^[16]^在胃癌中发现紧密连接蛋白Claudin-4、ZO-1与肿瘤侵袭性存在相关性。但是癌组织中ZO-1 mRNA在是否侵犯胸膜组别中却无明显差异，这可能是癌细胞在肺组织内扩散和淋巴转移略有不同，实体组织内肿瘤扩散不但和肿瘤侵袭性及肺内部微环境等有关，还和癌组织原发部位及病程长短有关。癌组织中ZO-1 mRNA在2年内死亡组和2年生存组中的差异具有统计学意义（*P*＜0.01），且癌旁组织中也存在显著统计学意义（*P*＜0.01），这意味着癌组织和癌旁组织中ZO-1 mRNA可能是一个和MA-15-5抗原^[17]^类似并与NSCLC 2年生存率密切相关的指标，但是否能作为临床预后的决定性因素，还需要进一步研究。
